# Is ERAS effective and safe in laparoscopic gastrectomy for gastric carcinoma? A meta-analysis

**DOI:** 10.1186/s12957-018-1309-6

**Published:** 2018-01-26

**Authors:** Ming-zhe Li, Wen-hui Wu, Liang Li, Xue-fu Zhou, Heng-liang Zhu, Jian-feng Li, Yu-long He

**Affiliations:** Department of Gastrointestinal Surgery, The Seventh Affiliated Hospital, Sun Yat-sen University, No.628 Zhenyuan Road, Guangming new district, Shenzhen, 518017 China

**Keywords:** Fast-track surgery, Gastric carcinoma, Laparoscopic, Meta-analysis

## Abstract

**Background:**

It is still unclear whether enhanced recovery after surgery is effective and safe in laparoscopic gastrectomy for gastric carcinoma.

**Methods:**

Cochrane library databases, Medline, Embase, and Pubmed were searched from January 1, 1986, to December 31, 2016. Randomized controlled trials (RCTs) comparing fast-track recovery with conventional recovery strategies in laparoscopic radical gastrectomy for gastric carcinoma were included. The main outcomes measured were postoperative hospital stay, time to first flatus, hospital charge, and overall complication rate.

**Results:**

Six RCTs with 400 patients were included in this study. Fast-track surgery has shorter postoperative hospital stays (weighted mean difference (WMD) − 2.65; 95% CI, − 4.01 to − 1.29, *z* = 3.82, *P* < 0.01) and less hospitalization expenditure (WMD − 523.43; 95% CI, − 799.79 to − 247.06, *z* = 3.71, *P* < 0.01) than conventional recovery strategies. There was no significant difference with respect to duration to first flatus (WMD − 17.72; 95% CI, − 39.46–4.02, *z* = 1.60, *P* = 0.11) and complication rate (OR 1.57; 95% CI, 0.82–2.98, *z* = 1.37, *P* = 0.17).

**Conclusions:**

Enhanced recovery after surgery is effective and safe and is thus recommended in laparoscopic radical gastrectomy for gastric carcinoma.

## Background

Gastric carcinoma is the second leading cause of cancer related deaths and is also the fourth most common carcinoma in the world [1]. Surgery is the most effective approach for treatment of gastric carcinoma. Laparoscopic gastrectomy was increasingly being used for gastric carcinoma in recent years. Compared with conventional surgery, laparoscopic gastrectomy had many advantages such as improvement in quality of life and faster recovery [2–4].

The enhanced recovery after surgery (ERAS), also called fast-track surgery (FTS), was initiated by Kehlet et al. in 1995 [5]. ERAS was a multidisciplinary approach aiming to accelerate recovery, minimize hospital stay, and reduce hospitalization expenditure, without compromising effectiveness and patient safety. Several randomized controlled trials (RCTs) and retrospective studies had documented the benefits and safety of FTS program implementation in laparoscopic radical gastrectomy for gastric carcinoma [6–12]. However, it was still unclear whether enhanced recovery after surgery is effective and safe in laparoscopic gastrectomy. Some studies comparing FTS and conventional recovery strategies in laparoscopic gastrectomy for gastric carcinoma gave conflicting results [13–18]. Therefore, the aim of this meta-analysis was to review the literature systematically and to investigate the safety and efficacy of FTS in laparoscopic gastrectomy for gastric carcinoma.

## Methods

### Search and selection strategies

This study was approved by the ethic committee of the Seventh Affiliated Hospital of Sun Yat-sen University. Two authors (Ming-zhe Li and Wen-hui Wu) performed a systematic article search independently. Cochrane library databases, Medline, Embase, and Pubmed between January 1986 and December 2016 were electronically searched. The medical subject headings were as follows: “accelerated rehabilitation,” “enhanced recovery,” “fast track,” “multimodal perioperative care,” “laparoscopy,” “laparoscopic,” “minimally invasive,” “gastrectomy,” “gastr*,” “gastric resection,” and “stomach.” The potentially relevant literature was retrieved through the consensus of the authors. The reference lists were cross-searched to identify additional literature. No language restriction was applied.

### Inclusion criteria and exclusion criteria

Randomized clinical trials that compared fast-track surgery with conventional recovery strategies for patients undergoing laparoscopic gastrectomy and were published in full were included. Trials should report at least one relevant outcome (postoperative hospital stay, duration to first flatus after operation, hospitalization expenditure, and short-term complication).

Authors with more than one published literature were represented by the most recent publication. Non-randomized studies, cohort studies, retrospective studies, and other studies that did not fulfill the inclusion criteria were excluded. Because this study focused only on laparoscopic gastrectomy, completely open or hand-assisted surgery was not included.

### Outcome measures

The outcome measures were postoperative hospital stay, duration to first flatus, hospital charge, and overall complication rate. Postoperative hospital stay was calculated from the date of surgery to the date of discharge.

### Method of review

This study was prepared in accordance with the Preferred Reporting Items for Systematic Reviews and Meta-Analyses (PRISMA) statement [19]. Each article was independently reviewed by two researchers (Ming-zhe Li and Wen-hui Wu) using the double method for eligibility by the inclusion criteria. Prior to final analysis, the article was confirmed by a third researcher (Yu-long He) and all conflicts were discussed and resolved. The quality of the randomized clinical trials was independently assessed using Jadad’s scoring system by two researchers (Ming-zhe Li and Wen-hui Wu) [20].

### Statistical analysis

Statistical analyses were performed using RevMan 5.0 software (Cochrane Information Management System). For continuous variables (postoperative hospital stay, duration to first flatus after surgery, and hospital charge), weighted mean differences (WMDs) with 95% confidence intervals (CIs) were calculated. For dichotomous variables (complication), odds ratios (ORs) with 95% CIs were calculated. In accordance with the current exchange rate, the exchange rate of the renminbi (RMB) against the US dollar in hospitalization expense is 6.879:1. *I*^2^ values were used to assess heterogeneity between articles. Publication bias was evaluated using funnel plots. If there was no significant heterogeneity among the studies, fixed effects model was used to pool studies. Otherwise, a random effects model was used. Level of statistical significance was set at *P* < 0.05.

## Results

### Article search

**Fig. 1:**
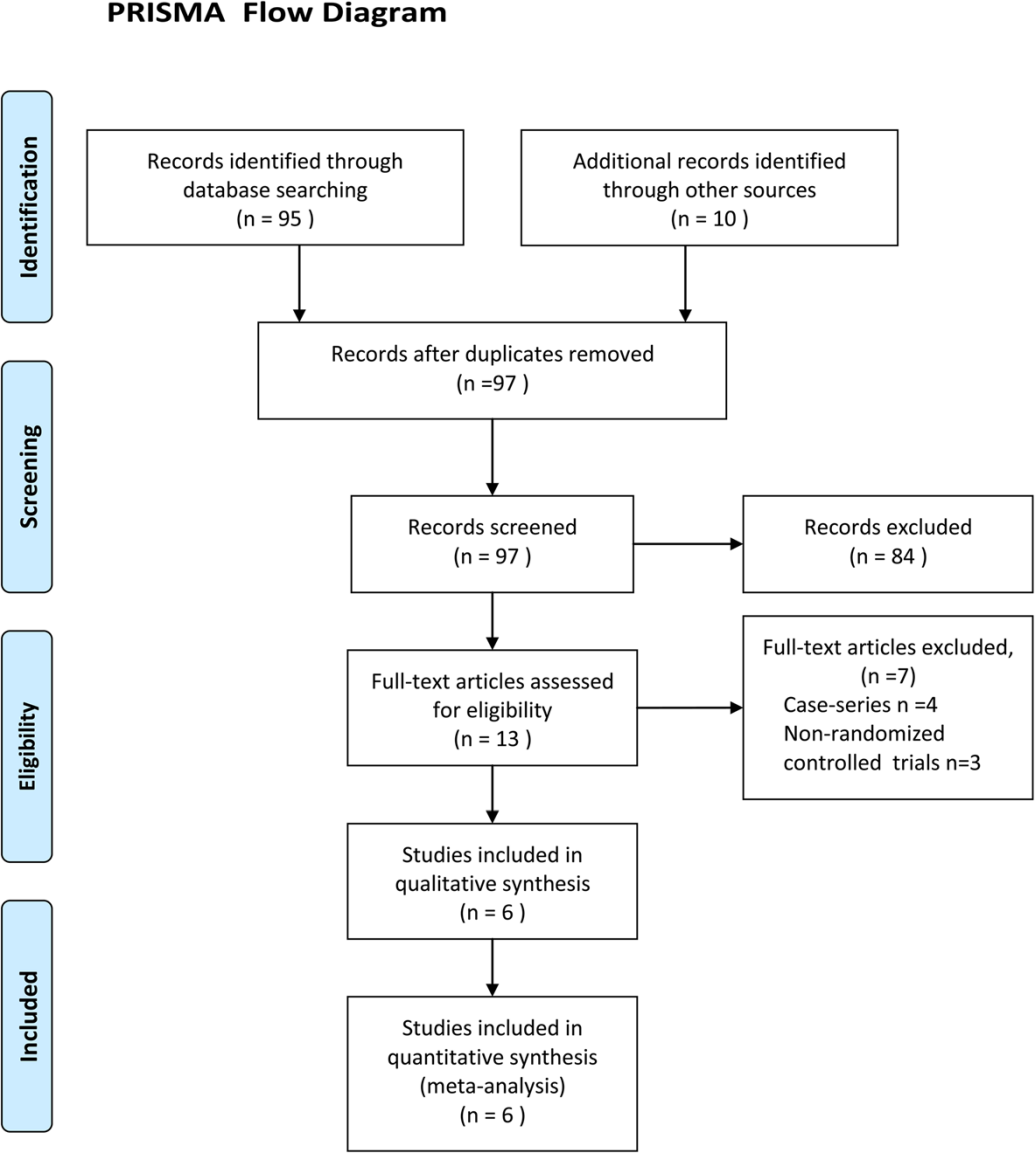
Article selection flow chart

One hundred and five articles were found by the literature search. Ninety-seven articles were selected for more detailed review after eliminating duplicates. Based on the inclusion criteria, 6 RCTs with 400 patients were included for meta-analysis (Fig. 1) [13–18]. Characteristics of each trial are shown in Table 1. Included literature was published from year 2012 to 2016. The number of patients in these studies ranged from 19 to 76. A total of 198 patients were in the FTS group, and 202 patients were in the conventional care group. The outcome variables (postoperative hospital stay, duration to first flatus after surgery, hospitalization expenditure, and short-term complication) extracted from these studies are presented in Table 2. Two studies did not report duration to first flatus after surgery, and three studies did not report hospital charge [15, 17, 18].

**Table 1:** Characteristics of studies included in this meta-analysis

Author	Year	Sample size FTS/conventional	Age (years) FTS/conventional	Gender (*n*, M:F) FTS/conventional	BMI (kg/m2) FTS/conventional	Type of surgery (*n*)
Hu et al. [13]	2012	19/22	59/62.5(median)	(10:9)/(10:12)	22.94 ± 2.23/22.99 ± 2.24	Distal gastrectomy(41)
Kim et al. [14]	2012	22/22	52.64/57.45(mean)	(13:9)/(15:7)	23.40 ± 3.17/23.77 ± 3.54	Distal gastrectomy(44)
Abdikarim et al. [15]	2015	30/31	63/62(median)	(21:9)/(20:11)	NR	Distal gastrectomy(44)/total gastrectomy(17)
Liu et al. [16]	2016	21/21	69.2/70.3(mean)	(10:11)/(12:9)	21.5 ± 2.0/21.9 ± 2.3	Proximal gastrectomy (10)/distal gastrectomy(21)/total gastrectomy(11)
Fang et al. [17]	2016	33/30	61.12/61.53(mean)	(15:18)/(16:14)	NR	NR
Xia et al. [18]	2016	73/76	61/63(median)	(48:25)/(50:26)	NR	NR

*FTS* fast-track surgery, *NR* not reported

**Table 2:** Outcome variables

Author	Postoperative hospital stay (days) FTS/conventional	First flatus after surgery (h) FTS/conventional	Hospital charge (dollars) FTS/conventional	Complication *N* (%) FTS/conventional
Hu et al. [13]	7(range 5.5–10)/7.5(range 6–11)	58(range 35–72)/65.5(range 35–72)	4815.51 ± 334.02/5212.72 ± 447.26*	12(63.2)/8(36.4)
Kim et al. [14]	5.36 ± 1.46/7.95 ± 1.98*	63.05 ± 18.62/67.41 ± 15.28	7454.3 ± 705.8/7771.8 ± 934.2	3 (13.6)/4 (18.2)
Abdikarim et al. [15]	6.8 ± 1.1/7.7 ± 1.1*	NR	NR	1(3.3)/2(6.5)
Liu et al. [16]	6.3 ± 1.5/7.8 ± 1.8*	48 ± 28.8/60 ± 26.4	4884.43 ± 407.04/5625.82 ± 276.2*	11(52.4)/6(28.6)
Fang et al. [17]	11.0 ± 2.0/18.5 ± 5.5*	60 ± 12/96 ± 24*	NR	2(7.1)/2(6.5)
Xia et al. [18]	6.38 ± 2.04/8.62 ± 2.87*	NR	NR	2(2.7)/2(2.6)

*FTS* fast-track surgery, *NR* not reported

*P < 0.05.

Italics indicate significance

### Methodological quality of included literature

**Fig. 2:**
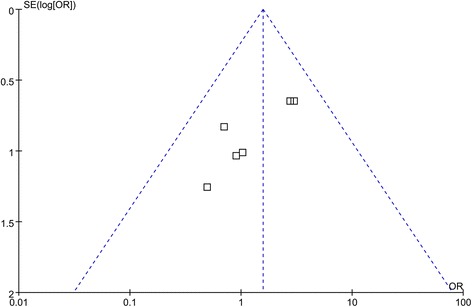
Funnel plot showing no significant publication bias

Regarding the methodological quality of the literature, all six studies were moderate to good (mean quality score 4) (Table 3). Randomization methods were reported in all six trials. Because of the design of the FTS program, most studies did not use blinding methods, except one study which used blinding of assessors [13]. No publication bias between the studies was observed (Fig. 2).

**Table 3:** Jadad’s score

Author	Year	Randomization	Allocation concealment	Blinding	Withdrawal	Total Jadad score
Hu et al. [13]	2012	1	1	1	1	4
Kim et al. [14]	2012	2	2	0	1	5
Abdikarim et al. [15]	2015	1	1	0	1	3
Liu et al. [16]	2016	2	1	0	1	4
Fang et al. [17]	2016	1	1	0	1	3
Xia et al. [18]	2016	2	2	0	1	5

### Postoperative hospital stay

**Fig. 3:**
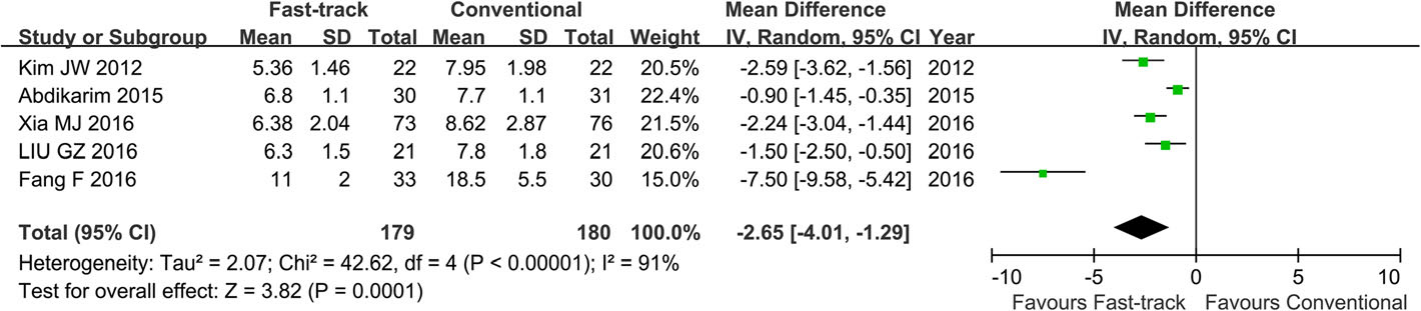
Forest plot describing postoperative hospital stay between FTS and conventional recovery strategies in laparoscopic gastrectomy for gastric cancer. FTS fast-track surgery

All six studies reported postoperative hospital stay [13–18]. One study reported medians and ranges instead of means and standard deviation (SDs) [13]. So, it could not be used in the meta-analysis. There was significant heterogeneity among the studies (*I*^2^ = 91%, *P* < 0.01) [14–18]. In random effects models, patients in the FTS group had a postoperative hospital stay of 2.65 days less than those in the conventional group (WMD − 2.65; 95% CI, − 4.01 to − 1.29, *z* = 3.82, *P* < 0.01; Fig. 3).

### Duration to first flatus after surgery

**Fig. 4:**
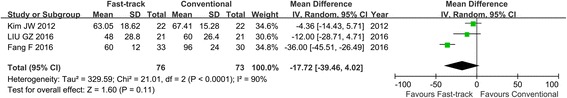
Forest plot describing duration to first flatus after surgery between FTS and conventional recovery strategies in laparoscopic gastrectomy for gastric cancer. FTS fast-track surgery

Only three studies reported duration to first flatus after surgery [14, 16, 17]. There was significant heterogeneity among the studies (*I*^2^ = 90%, *P* < 0.01). In random effects models, there was no significant difference in duration to first flatus after surgery between that of the FTS group and conventional group (WMD − 17.72; 95% CI, − 39.46–4.02, *z* = 1.60, *P* = 0.11; Fig. 4).

### Hospital charge

**Fig. 5:**
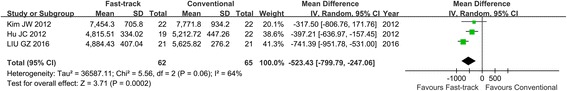
Forest plot describing hospital charge between FTS and conventional recovery strategies in laparoscopic gastrectomy for gastric cancer. FTS fast-track surgery

Only three studies reported hospital charge [13, 14, 16]. There was significant heterogeneity among the studies (*I*^2^ = 64%, *P* = 0.06). In random effects models, patients in the FTS group had a hospital charge of 523.43 dollars less than those in the conventional group. (WMD − 523.43; 95% CI, − 799.79 to − 247.06, *z* = 3.71, *P* < 0.01; Fig. 5).

### Overall complication rate

**Fig. 6:**
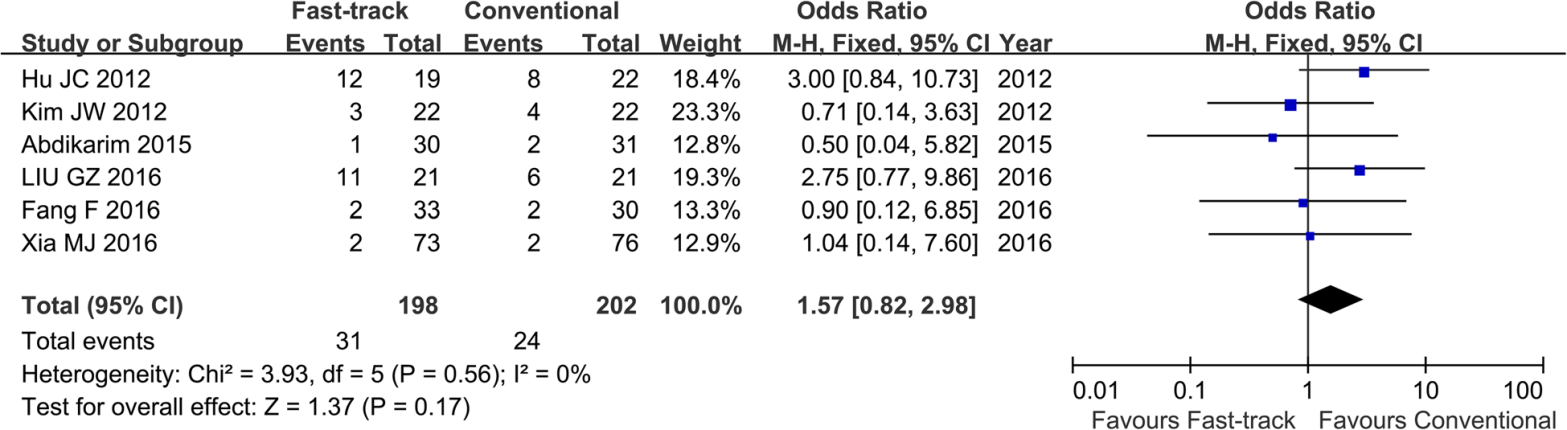
Forest plot describing complication rate between FTS and conventional recovery strategies in laparoscopic gastrectomy for gastric cancer. FTS fast-track surgery

All six studies reported postoperative complication rate [13–18]. There was no significant heterogeneity among the studies (*I*^2^ = 0%, *P* = 0.56). In fixed effects models, there was no significant difference in complication rates between the FTS group and conventional group. (OR 1.57; 95% CI, 0.82–2.98, *z* = 1.37, *P* = 0.17; Fig. 6).

## Discussion

The fast-track surgery (FTS) consists of a multidisciplinary approach including patients’ education, anesthesia, goal-directed fluid therapy, prevention of nausea and ileus, temperature monitoring, early nutrition, and early mobilization. Many studies had demonstrated that FTS in D2 gastrectomy was safe and efficient, and it could reduce postoperative stresses [21]. Some meta-analysis also demonstrated the FTS protocol was feasible for gastric cancer patients who underwent gastrectomy. Li et al. found that compared with conventional care, FTS shortened the postoperative stay (WMD − 2.00; 95% CI − 2.69 to − 1.30, *z* = 5.64, *P* < 0.00001), and reduced hospitalization costs (WMD − 447.72; 95% CI − 615.92 to − 279.51, *z* = 5.22, *P* < 0.00001). There was no significant difference in postoperative complications (*P* = 0.07) [22]. Yu et al. showed that postoperative hospital stay, time to first passage of flatus, and hospital costs were significantly reduced for fast-track surgery. No significant differences were found for readmission rates and total postoperative complications [23]. All these meta-analyses focused on open gastrectomy for gastric cancer combined with FTS protocol. Little study focused on the safety and effectiveness in laparoscopic gastrectomy combined with FTS protocol. Recently, the advantages of laparoscopic gastrectomy had also been recognized. Some studies had demonstrated that laparoscopic gastrectomy could alleviate inflammation and immune inhibition and accelerate postoperative recovery [24–29]. However, there was little study comparing the effect of FTS program in laparoscopic gastrectomy, and the result was conflicting [13–18]. So, in the present study, we focused on four short-term outcomes and aimed to investigate the safety and efficacy of FTS in laparoscopic gastrectomy for gastric carcinoma.

Our study revealed postoperative hospital stay was significantly shorter in the FTS group than in the conventional group (Fig. 3). Both FTS and laparoscopic surgery could reduce the postoperative stress response and promote rehabilitation [30–32]. Therefore, combining the two methods would result in the fastest postoperative recovery. We also found there was a trend toward shorter time of first flatus for the FTS group. However, the result of the meta-analysis failed to show any difference, although the trend favored the FTS group (Fig. 4). The reason might be that the power of the test was weak. The sample size was too small to detect the real difference between the two groups. In our study, the hospital charge was significantly different between the two groups. The FTS group had lower medical costs than conventional group (Fig. 5), which might be explained by the effect of the fast-track recovery system.

With respect to complication rate, there was no significant difference in complication rates between the FTS group and conventional group (Fig. 6). In the present meta-analysis, the complication rates in each study were varied (2.63–63.2%). Many factors could influence the occurrence of postoperative complications, like surgeon’s surgical technique, perioperative care, and physical condition of patients. However, we found a trend toward higher complication rate for the FTS group in the present study (Fig. 6). We were not sure whether this was associated with perioperative FTS. Therefore, more studies were needed to confirm the safety of FTS in laparoscopic gastrectomy.

There were some limitations to this study. Firstly, the sample of RCTs was small. Therefore, the power of the test might be weakened and the real difference might not be apparent presently. Secondly, the studies showed significant heterogeneity in the outcome measures. This heterogeneity might be due to diversity in medical and economic status in different regions. Thirdly, only two studies included in the meta-analysis dealt solely with laparoscopic distal gastrectomy [13, 14]. The other four studies dealt with the laparoscopic proximal stomach, distal gastrectomy, and total gastrectomy. It was not possible to tease out these numbers. Therefore, we could not know whether the results will change if the total gastrectomy patients were separated from the distal gastrectomy patients. Last but not least, methodological quality of included studies was moderate. Most studies did not use blinding methods, except one study which used blinding of assessors. It was hard to use double-blind methods in most clinical trials, and therefore, this limitation was difficult to solve.

## Conclusions

This meta-analysis showed that fast-track perioperative care has shorter postoperative hospital stays and less hospitalization expenditure than conventional recovery strategies in laparoscopic gastrectomy. There is no significant difference with respect to complication rate. Fast-track surgery is safe and effective in laparoscopic gastrectomy for gastric carcinoma. However, the sample of RCTs included in the meta-analysis is small. Large-sample randomized controlled trials should be encouraged to ensure that the benefits of fast-track surgery can be applied to laparoscopic gastrectomy.

## Abbreviations

CI: Confidence intervals
ERAS: Enhanced recovery after surgery
FTS: Fast-track surgery
OR: Odds ratio
PRISMA: Preferred Reporting Items for Systematic Reviews and Meta-Analyses WMD Weighted mean difference
RCTs: Randomized controlled trials
SD: Standard deviation
